# Aiming for a representative sample: Simulating random versus purposive strategies for hospital selection

**DOI:** 10.1186/s12874-015-0089-8

**Published:** 2015-10-23

**Authors:** Loan R. van Hoeven, Mart P. Janssen, Kit C. B. Roes, Hendrik Koffijberg

**Affiliations:** Julius Center for Health Sciences and Primary Care, University Medical Center Utrecht, Universiteitsweg 100, 3584 CG Utrecht, The Netherlands; Sanquin Blood Supply, Transfusion Technology Assessment Department, Sanquin Research, Amsterdam, Universiteitsweg 100, 3584 CG Utrecht, The Netherlands; Department of Health Technology & Services Research, MIRA Institute for biomedical technology and technical medicine, University of Twente, Drienerlolaan 5, 7522 NB Enschede, The Netherlands

**Keywords:** Sampling strategy, Hospital selection, Representativeness, Random vs. purposive sampling, Maximum variation, Simulation, Model-based inference

## Abstract

**Background:**

A ubiquitous issue in research is that of selecting a representative sample from the study population. While random sampling strategies are the gold standard, in practice, random sampling of participants is not always feasible nor necessarily the optimal choice. In our case, a selection must be made of 12 hospitals (out of 89 Dutch hospitals in total). With this selection of 12 hospitals, it should be possible to estimate blood use in the remaining hospitals as well. In this paper, we evaluate both random and purposive strategies for the case of estimating blood use in Dutch hospitals.

**Methods:**

Available population-wide data on hospital blood use and number of hospital beds are used to simulate five sampling strategies: (1) select only the largest hospitals, (2) select the largest and the smallest hospitals (‘maximum variation’), (3) select hospitals randomly, (4) select hospitals from as many different geographic regions as possible, (5) select hospitals from only two regions. Simulations of each strategy result in different selections of hospitals, that are each used to estimate blood use in the remaining hospitals. The estimates are compared to the actual population values; the subsequent prediction errors are used to indicate the quality of the sampling strategy.

**Results:**

The strategy leading to the lowest prediction error in the case study was maximum variation sampling, followed by random, regional variation and two-region sampling, with sampling the largest hospitals resulting in the worst performance. Maximum variation sampling led to a hospital level prediction error of 15 %, whereas random sampling led to a prediction error of 19 % (95 % CI 17 %-26 %). While lowering the sample size reduced the differences between maximum variation and the random strategies, increasing sample size to *n* = 18 did not change the ranking of the strategies and led to only slightly better predictions.

**Conclusions:**

The optimal strategy for estimating blood use was maximum variation sampling. When proxy data are available, it is possible to evaluate random and purposive sampling strategies using simulations before the start of the study. The results enable researchers to make a more educated choice of an appropriate sampling strategy.

**Electronic supplementary material:**

The online version of this article (doi:10.1186/s12874-015-0089-8) contains supplementary material, which is available to authorized users.

## Background

When choosing a sample of participants, researchers often find themselves with a trade-off between the wish for randomization and pragmatic considerations. Random sampling is the gold standard of sampling strategies because of its unbiasedness and the possibility to evaluate the reliability (precision) of the resulting estimates [1–4]. Random sampling is not, however, always feasible in practice due to constraints in time, resources and costs, and researchers in the medical field often use a ‘convenience’ or a purposive sample, i.e. by choosing participants who are easy to recruit or by selecting participants based on preferences or expectations.

Fortunately, some studies suggest that such purposive strategies can lead to representative samples [5–7], where a sample is considered representative when either sample characteristics or inferences from the sample approximate population values. Also, the statement that a random sample is unbiased means that it will provide a representative estimate on average. The probability of randomly drawing an ‘unrepresentative’ sample is large if your population is small; the estimator is not robust, since data collection is done only once and not a thousand times. This can be illustrated by a study in the medical field that compared a randomized study design with a nonrandomized design. The nonrandomized design resulted in a more representative sample in 34 % of cases [5]. In another study, comparison of several sampling strategies for the surveillance of cases of injury and poisoning in accident and emergency departments showed that a well-planned systematic sampling strategy can generate data of equal quality to surveillance including all patients [7]. In a study estimating drug use characteristics, purposive samples were found to be sufficiently representative, as compared to probabilistic strategies, when these were drawn from a wide cross-section of participants and included a relatively large number of individuals [6]. Thus non-probabilistic strategies are sufficient at least in some cases.

If possible, strategies should be evaluated per study, in line with the ‘fit for use’ concept; see [8,9]). Preferably this evaluation should be done prior to the actual data collection so that this information can be used to choose the optimal sampling strategy. However, in the medical field, to our knowledge, no (simulation) studies exist that evaluate random versus preferential sampling strategies with respect to prediction accuracy before data collection; instead, methods exist for generalizing treatment effects in randomized trials from unrepresentative samples (see for example [5,10–12]), or studies that either compare only non-probabilistic [13,14] or only probabilistic strategies [15].

In the present study, in order to find the optimal strategy, we compare five stratified probabilistic (i.e., containing a random element) and purposive (more broadly: non-probabilistic) sampling strategies that match real-life strategies used in practice. The goal is to find the strategy that results in the most representative sample of hospitals, which in this paper we define as a sample that allows us to estimate blood use in the remaining hospitals with a prediction error as low as possible. The case is based on an actual ongoing study (named PROTON II; www.proton2.nl), in which we want to study optimal blood use and benchmark Dutch hospitals. The resulting database (containing data from 12 selected hospitals) will include detailed information on patient diagnoses and clinical parameters that can be used to answer several research questions concerning blood use, such as: ‘Which patient groups receive blood (and how much) in terms of patient age, diagnosis and surgical procedures?‘and “What are determinants and triggers of blood use?’. Ideally we would include all Dutch hospitals, but this is not possible since the process of extracting large amounts of data was found to be complicated and time-consuming, mainly due to variation between hospitals and the electronic information systems that they used. Therefore, a selection must be made of 12 hospitals (out of 89 in total).

For the simulation, we used a limited amount of data that was already available for each hospital before data collection. Five pragmatic sampling strategies were simulated (stratified to hospital type as stratification has been proven beneficial [16]): 1). Largest hospitals sampling (resulting in a large database), 2) Maximum variation sampling (only hospitals on the most extreme ends of red blood cell use are sampled), 3) Random sampling, 4), Regional variation sampling (hospitals from each region are included) and 5) Two geographic regions sampling. Representativeness of the resulting samples is evaluated by performing model based inference and computing the prediction errors [17]. We assume that if a sample is representative in this restricted dataset, it will also be representative for other relevant population outcomes. The results will show whether or not, in the context of estimating blood use, the general consensus that non-probabilistic strategies are inferior to probabilistic strategies holds. More broadly, the case illustrates a method for evaluating different sampling strategies. The results can be used to support an informed choice of sampling strategy.

## Methods

Five pragmatic sampling strategies (see below) are simulated using information that is already available prior to the actual data collection. The effect of sampling strategy is evaluated in terms of its accuracy in predicting the population estimates and the margin of error.

### Case and data

The target population consists of all non-specialized Dutch hospitals (*n* = 89), comprising 8 academic centres, 28 teaching hospitals and 53 remaining general (smaller) hospitals. Specialized centres were excluded (*n* = 3), because the majority of blood transfusions is already covered by the academic and peripheral hospitals. Limited data on all hospitals was already available and easily accessible. Firstly, the number of beds per hospital was extracted from annual hospital reports or the hospital website. Secondly, hospitals were classified by type, as described by Dutch Hospital Data [18]: a hospital is either an academic medical centre, teaching hospital or general hospital. Proxy information on hospital blood use was available as well. Sanquin Blood Bank granted permission for accessing data on the number of issued blood products delivered to each hospital in the year 2013 for the three main product types: red blood cell products (RBC), fresh frozen plasma products (FFP) and platelet products (PLT) [19]. Requests for access to these data may be addressed to the Vice Chair Executive Board of Sanquin. The number of issued blood products was used as a proxy for the number of transfused blood products. If information on blood use was not available for a hospital, that hospital was excluded from the analysis (*n* = 1). Classification of hospitals into organizational healthcare regions was done according to the Education and Research regions [20]; hospitals that were not classified by this structure (*n* = 8) were manually assigned based on their location to the nearest region.

### Sampling strategies

**Fig. 1:**
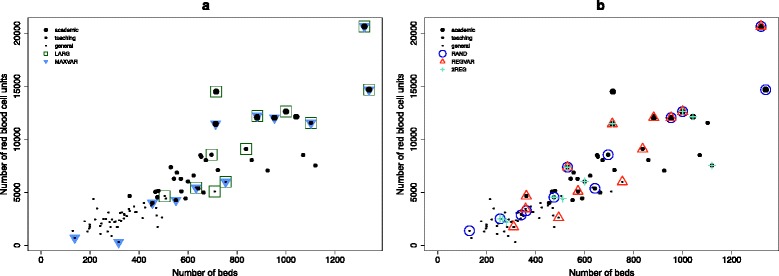
Illustration of sampling strategies. **a** Hospitals that are selected when using the non-probabilistic strategies of sampling the larges hospitals (LARG) and maximum variation (MAXVAR); **b** Possible selection of hospitals when the probabilistic RAND, REGVAR and 2REG strategies are conducted (since these strategies involve a random element, the figure shows only one of many possible samples)

The simulation is confined to the following five strategies, which are all stratified to hospital type with a fixed sample size ratio of 1:1:1 of the strata: 1) Maximum variation sampling (MAXVAR) is used to sample hospitals that have the highest and lowest number of RBC transfusions, so that variation in the total number of RBCs is maximized. The theoretical advantage of maximum variation sampling is that extrapolating to extreme (impossible) values does not occur because the extremes are already in the sample. Selecting hospitals based on their RBC use is also supposedly sufficient for obtaining high variation in FFP and PLT use; the respective Spearman’s rank correlations with RBC use are .88 (p < .00001) and .92 (p < .00001). 2) Sampling only the largest hospitals (LARG) has the obvious advantage that since larger hospitals have more patients, this yields the most data. 3) Random sampling (RAND) gives each hospital within a stratum an equal probability of being sampled. 4) Regional variation sampling (REGVAR) maximizes the number of randomly chosen organizational health care regions (*n* = 7) included for each stratum, based on the assumption that there is considerable variation between regions that must be reflected in the sample. 5) Sampling from two organizational health care regions (2REG). Including a large part of all hospitals from two regions allows not only the benchmarking of hospitals, but also the benchmarking of (almost) complete regions. This form of sampling is simulated for all 21 combinations of two regions. If a region contains more hospitals than the preferred sample size per stratum, hospitals are selected randomly. In contrast, if a region contained fewer hospitals than the preferred sample size within a stratum, all hospitals within that stratum are included. Figure 1a illustrates which hospitals are sampled when using the purposive strategies LARG and MAXVAR. Figure 1b shows a possible result of sampling when the RAND, REGVAR and 2REG strategies are used. The RAND, REGVAR and 2REG strategies can all be classified as probabilistic or as they contain a random component, and will collectively be referred to as ‘the random strategies’.

### Sample size

Sample size is varied, starting with four hospitals per stratum (n total = 12). In each subsequent scenario the added value of including two more of each type of hospital (thus 6 hospitals per stratum) is examined, as well as the effect of including two hospitals fewer in each stratum (see Table 1 for all three sample size scenarios). The stratification by hospital type ensures a fixed sample size ratio of the strata of 1:1:1. The exception is the strategy of sampling two regions; here, since there is usually only one academic hospital per region, the number of included academic hospitals is fixed in all scenarios at two (and three when the region Noord-Holland is included).

**Table 1:** Number of hospitals included per type for each sample size scenario

Scenario	*N* (academic)	*N* (teaching)	*N* (general)	*N* Total (% of all Dutch hospitals)
A	4	4	4	12 (13 %)
B	6	6	6	18 (20 %)
C	2	2	2	6 (7 %)

### Model-based inference

Model-based inference, since it may provide a viable alternative for design-based methods when design information is not available [21], seems most appropriate for non-probabilistic sampling. Even if a non-probabilistic sample in itself is not representative, the resulting model might very well be [22]. In the present case, model based inference was used to predict hospital blood use. A Poisson distribution was fitted because the outcomes are count data, and the association between number of beds and number of products, when stratified to hospital type, seems exponential. In short, a data sample drawn according to one of the above strategies was used to fit a Poisson regression model that predicts blood use (i.e., the number of RBC, FFP and PLT per hospital) as a function of hospital size (i.e., number of beds) and hospital type. With the obtained prediction models, RBC, FFP and PLT use is estimated for all Dutch hospitals and compared to the true population values, which are known for this case. For each of the three blood products, outcomes are: 1) the hospital level prediction error and 2) the national level prediction error. For the hospital level error, the absolute prediction error of the number of products is calculated for each hospital. These errors are summed over all hospitals and the resulting ‘absolute error’ is expressed as a percentage of the total number of products, i.e. the population value. For the national level prediction error, the predictions for all hospitals are summed, resulting in an estimate of the total number of products. The difference between this ‘national estimate’ and the actual population value is the ‘national error’, which is also expressed as a percentage of the population value. These two error types are of interest for different reasons. National level errors are important from the perspective of the national blood bank: since the blood bank produces blood products for the whole of the Netherlands, it is relevant to know how much blood is needed in total, for example on a yearly basis. However from the perspective of (clinical) studies, it might be considered more important to have accurate predictions also within each hospital (i.e., at the hospital level). Obviously, individual hospitals are also interested in their own expected blood use.

### Simulations

For each of the RAND, REGVAR and 2REG strategies, a random sampling process is simulated a thousand times. The median error percentages are reported, with the 95 % centiles and the average error percentages. Since the strategy of sampling two regions encompasses 21 unique combinations of two regions, the median and average error percentages are taken over all sampled combinations. Striking differences between combinations of regions are described in the results section. All analyses are performed in R Version 3.0.0.

## Results

### Prediction error at the hospital level

**Fig. 2:**
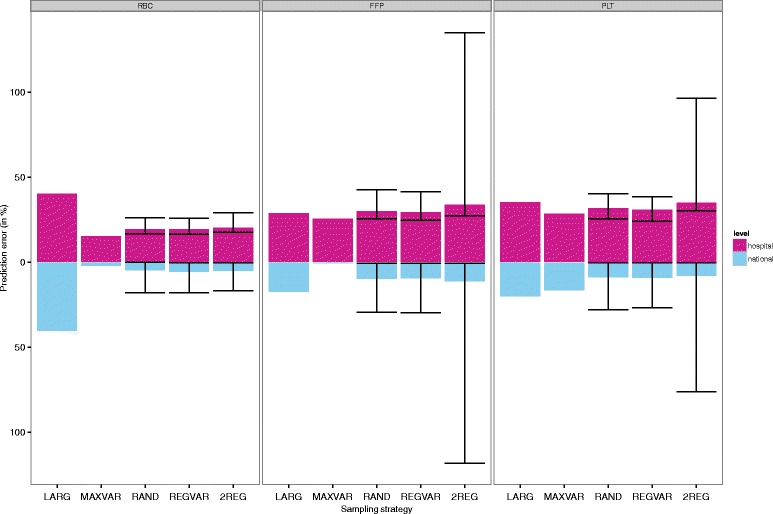
Prediction error at hospital and national level for n(academic) = 4, n(teaching) = 4 and n(general) = 4. Median prediction errors of red blood cell (RBC), plasma (FFP) and platelet (PLT) use, for different sampling strategies. 95 % centiles are provided for the strategies involving a random element. Number of simulations = 1000. LARG = largest hospitals, MAXVAR = maximum variation in number of RBCs, RAND = random, REGVAR = regional variation, 2REG = two regions

Prediction errors for each sampling strategy are shown in Table 2, for the scenario of sampling 12 hospitals. For RBC, FFP and PLT, maximum variation sampling outperformed largest hospitals sampling in terms of hospital level error (Fig. 2). Similarly, MAXVAR consistently outperformed the random strategies (RAND, REGVAR and 2REG). MAXVAR sampling resulted in a 15 % prediction error at the hospital level for RBC, whereas the random strategies (stratified random, regional variation and two regions) had a slightly higher median error for RBC (namely 19 %, 19 %, and 20 % respectively), for FFP (30 %, 29 % and 34 % versus 25 % for MAXVAR), and for PLT (32,31 % and 35 % versus 28 % for MAXVAR). Of all the simulations, RAND resulted in a lower hospital level error than MAXVAR in none (RBC), 3 % (FFP) and 14 % (PLT) of the simulations (Table 3). Sampling only the largest hospitals resulted in a hospital level error of 40 % for RBC, which is higher than the median errors for the random strategies.

**Table 2:** Comparison of prediction errors for the five sampling strategies, for *n* = 12 (*n* = 4 per hospital type)

Strategy	Prediction error at hospital level			Prediction error at national level		
	*RBC*	*FFP*	*PLT*	*RBC*	*FFP*	*PLT*
LARG	40 %	28 %	35 %	40 %	17 %	20 %
MAXVAR	15 %	25 %	28 %	2 %	1 %	17 %
RAND (median; mean, 95 % centiles)	19 %; 20 % (17 %-26 %)	30 %; 31 % (25 %-42 %)	32 %; 32 % (25 %-40 %)	5 %; 6 % (0 %-18 %)	10 %; 11 % (1 %-30 %)	9 %; 10 % (0 %-28 %)
REGVAR (median; mean, 95 % centiles)	19 %; 20 % (16 %-26 %)	29 %; 30 % (25 %-41 %)	31 %; 31 % (24 %-39 %)	6 %; 7 % (0 %-18 %)	9 %; 11 % (1 %-30 %)	9 %; 10 % (0 %-27 %)
2REG (median; mean, 95 % centiles)	20 %; 21 % (17 %-29 %)	34 %; 49 % (27 %-135 %)	35 %; 44 % (30 %-43 %)	5 %; 6 % (0 %-17 %)	11 %; 26 % (1 %-118 %)	8 %; 16 % (0 %-76 %)

LARG = largest hospitals, MAXVAR = maximum variation in number of RBCs, RAND = random, REGVAR = regional variation, 2REG = two regions, RBC = red blood cell products, FFP = fresh frozen plasma products, PLT = platelet products. Output for RAND, REGVAR and 2REG is based on the average of 10 times 1000 simulations and accompanied by 95 % centiles. Outcomes are the prediction error at hospital level (summed absolute errors at hospital level) and the national prediction error (absolute deviation of the national estimate from the population values), both expressed as a percentage from the population values

**Table 3:** How often are predictions of blood use better for random than for purposive sampling strategies?

RAND versus:	MAXVAR			LARG		
	*RBC*	*FFP*	*PLT*	*RBC*	*FFP*	*PLT*
Lower hospital level prediction error for RAND	0 %	3 %	14 %	100 %	35 %	84 %
Lower national level prediction error for RAND	25 %	3 %	79 %	100 %	81 %	87 %
REGVAR versus:	MAXVAR			LARG		
Lower hospital level prediction error for REGVAR	0 %	6 %	25 %	100 %	44 %	90 %
Lower national level prediction error for REGVAR	20 %	4 %	79 %	100 %	79 %	88 %
2REG versus:	MAXVAR			LARG		
Lower hospital level prediction error for 2REG	0 %	0 %	0 %	100 %	9 %	52 %
Lower national level prediction error for 2REG	24 %	3 %	83 %	100 %	70 %	88 %

Percentage of all simulations that the random strategies (RAND, REGVAR and 2REG) outperform the purposive strategies (MAXVAR and LARG) in terms of hospital and national level prediction error for *n* = 12 (*n* = 4 per hospital type)

### Prediction error at the national level

Comparing the national level prediction errors of the strategies resulted in a similar pattern, with MAXVAR marginally but consistently outperforming the random strategies most of the time (Table 2 and Fig. 2). In fact, random sampling resulted in a lower national level error than MAXVAR in 25 % (RBC), 3 % (FFP) and 79 % (PLT) of the simulations (Table 3), with PLT being the exception. The same pattern was found for REGVAR and 2REG.

### Effect of sample size

Increasing the sample size did not affect the overall ranking of the sampling strategies by prediction error; the scenarios *n* = 12, *n* = 18 and *n* = 6 all resulted in a preference for MAXVAR. Adding two more academic, two more teaching and two more general hospitals to the sample (total *n* = 18) reduced hospital and national level error as well as the 95 % percentile error ranges by approximately two absolute percent points, but not always (see Additional file 1). Decreasing the sample size to two hospitals per hospital type (total *n* = 6) reduced the difference between MAXVAR and the random strategies. Also, decreasing sample size increased prediction errors considerably in some cases, especially for the LARG strategy. For LARG, both hospital and national level error for RBC increased from 40 % to 92 %. For MAXVAR, hospital level errors increased from 15 % to 23 % (RBC), 25 % to 37 % (FFP) and 28 % to 40 % (PLT); national level errors increased by three percent points. Similarly, the random strategies yielded moderately higher hospital and national errors and wider 95 % centile ranges in the low sample size scenario (see Additional file 2).

## Discussion

Currently, the representativeness of a sample is often only checked after data collection has finished. Although such a post-hoc evaluation may provide some insight into the representativeness of the already selected sample, unfavourable outcomes can rarely be mitigated once the data collection process has ended. Therefore an evaluation of potential sampling strategies should ideally be performed prior to data collection. We evaluated five pragmatic sampling strategies for the case of estimating blood use in Dutch hospitals. The evaluation consists of simulating the sampling processes for five probabilistic and non-probabilistic strategies, using prior knowledge. Such a simulation study may help in deciding whether a random sampling design is necessary or whether variation should be aimed for in order to obtain a sample that is likely to be representative of population values. This type of evaluation is in theory applicable to a broad array of research fields, provided that there exists an association between a predictor and outcome that can be modelled and data are available before the actual study.

The case study illustrates that random sampling, which is considered the gold standard, is not necessarily the optimal sampling strategy. In fact, of the five strategies considered, the optimal strategy in our case was maximum variation sampling (MAXVAR). A sample selected using the MAXVAR strategy led to better predictions of red blood cell unit (RBC) use than a random sample in all (at the hospital level) or most (at the national level) of the simulations. In contrast, random sampling did perform much better than sampling only the largest hospitals. In general, the same pattern was found for both national and hospital level prediction errors, with national errors being lower since under- and overestimation of individual hospitals cancel each other out. Differences between MAXVAR and the random strategies appear small but consistent.

The preference for the non-probabilistic MAXVAR strategy over random sampling was not completely expected in the context of previous literature. In a previous study that simulated outcomes of a randomized design [5], non-probabilistic sampling was reported to be better in only 34 % of simulations. Moreover, in a study on modelling species distribution, non-probabilistic strategies were reportedly inferior to probabilistic strategies [23]. These contrasting findings could be due to the use of different measures for evaluating representativeness: in the present study a sample is considered representative if it gives us an unbiased estimate of the outcome studied, whereas in other studies, representativeness is defined in terms of whether participants in the sample have similar characteristics as those in a random sample. Moreover, these contrasting findings could be caused by the use of different data and models for inference, and the use of a convenience sample instead of systematic purposive samples as in the present study. However, in line with earlier findings [5], differences between the median prediction errors for MAXVAR and the random strategies were quite small. This implies a trade-off between the certainty of a known prediction error with MAXVAR and the risk of potentially getting a higher (or a lower) error with one of the random strategies.

In the present study, MAXVAR seems the ‘safest’ option. However, MAXVAR was not consistently the preferred strategy. In accordance with findings from an ecological study [16], preference for either a non-probabilistic or a probabilistic strategy turned out to depend on which outcome was modelled. For example, MAXVAR outperformed random sampling for the outcome platelet (PLT) use at the hospital level, but not at the national level. Apparently for national level predictions of PLT use, MAXVAR results in a systematic deviation (i.e., predictions per hospital do not cancel each other out). This result might be explained by differences in the underlying distributions of the outcomes predicted. Compared to RBC use, the distribution of PLT use has more extreme values, as PLT use is relatively high in the largest hospitals and varies greatly between hospitals (partly due to its high spilling rate and short shelf life). In case the outcome distribution does not have values that are too extreme or when the association between predictor and outcome is more or less linear, MAXVAR seems to perform well. However for other distributions, an amended MAXVAR strategy might be more suitable. Such a strategy could for instance maximize the distances between subsequent hospitals (instead of sampling at the ends of the distribution). Choosing an appropriate variable for which maximum variation is obtained is important, since this can have substantial consequences for the estimates [16]. Ultimately, whether prediction accuracy at the hospital or at the national level is given more importance depends on the aim and perspective of the researcher. From the perspective of the blood bank that produces blood products for the whole of the Netherlands, it is important to know how much blood is needed nationally on a yearly basis. However from the perspective of (clinical) studies and individual hospitals, it would be more important to have accurate predictions also within each hospital.

Increasing the sample size from 12 to 18 hospitals did not alter the order of the strategies. In contrast, decreasing the sample size to 6 hospitals diminished the advantage of MAXVAR sampling over the random strategies. Apparently a very low sample size in MAXVAR sampling allows outlier hospitals to impact the model estimates more heavily, leading to relatively high prediction errors. In comparison, in a study on habitat suitability modelling [24], increasing or decreasing sample size did not change the order of strategies, however that study did not consider scenarios with a sample size as small as *n* = 6. In that study, prediction accuracy increased with sample size, whereas in our study the gain in prediction accuracy for *n* = 18 as compared to *n* = 12 was quite small (around 1–2 absolute percentage points). Presently, for estimating blood use it is not directly obvious that including six additional hospitals would be worth the additional effort. Instead, a more accurate prediction of blood use might have been obtained by extending the model with more detailed information (predictors), such as the presence of a cardiac centre in a hospital and number of patients per admission diagnosis or type of surgery.

An important assumption that underlies our evaluation is that representativeness in terms of a known outcome can be used as a proxy for representativeness for other outcomes (which will be studied after the actual data collection). The reasoning behind this assumption is that if a sample is at least representative for the number of blood products, it is more likely to be representative for related outcomes as well. These related outcomes, such as the distribution of blood products over diagnoses and surgeries, blood use in different patient subgroups, and transfusion triggers, are all expected to be related to the predictors hospital type and hospital size. A second assumption made is that a limited nationwide data set is available. If no such proxy data would be available, an option would be to simulate the data set as well. Further research could investigate what the optimal sampling strategy would be for different assumptions about the distributions and associations within the data. Finally, we acknowledge that other considerations such as costs, feasibility, the need to include specific regions for benchmarking purposes, specific patient groups, or certain hospitals from which historical data are already available (which enables a trend analysis), might play a role in selecting potential participants. These conditions can also be included in the simulation. Last but certainly not trivial, the success of the data selection depends on the cooperation of the potential participants.

## Conclusion

A simulation study as described above may offer guidance in choosing an appropriate sampling strategy and size before data collection is started. Following this guidance is straightforward and can be done with limited resources. Its only requirement is the a priori availability of a (limited) nationwide data set, which will often be available as long as the aggregation level is sufficiently high. In many situations, especially whenever data collection has large resource requirements, such a simulation will be worthwhile and should therefore be considered.

## Availability of data and materials

Availability of the data used is not necessary as not the data itself but the simulation method is of interest. Instead we provide the R code that creates an exemplary data set and simulates the sampling strategies (Additional file 3).

## Additional files

Additional file 1:
**Comparison of prediction errors for the five sampling strategies, for** ***n*** **= 18** **(** ***n*** **= 6 per hospital type).** File legend: LARG = largest hospitals, MAXVAR = maximum variation in number of RBCs, RAND = random, REGVAR = regional variation, 2REG = two regions, RBC = red blood cell products, FFP = fresh frozen plasma products, PLT = platelet products. Output for RAND, REGVAR and 2REG is accompanied by 95 % centiles. Description: These show the prediction errors for *n* = 18 in a similar table as for *n* = 12. (XLSX 10 kb) Additional file 2;
**Comparison of prediction errors for the five sampling strategies, for** ***n*** **= 6** **(** ***n*** **= 2 per hospital type).** File legend: LARG = largest hospitals, MAXVAR = maximum variation in number of RBCs, RAND = random, REGVAR = regional variation, 2REG = two regions, RBC = red blood cell products, FFP = fresh frozen plasma products, PLT = platelet products. Output for RAND, REGVAR and 2REG is accompanied by 95 % centiles. Description: These show the prediction errors for *n* = 6 in a similar table as for *n* = 12. (XLSX 9 kb) Additional file 3:
**R code for simulating sampling strategies.** Description: R code that creates an exemplary data set and simulates the sampling strategies. (R 26 kb)

## Abbreviations

RBC: Red blood cell unit
FFP: Fresh frozen plasma unit
PLT: Platelet unit
LARG: Strategy of sampling only the largest hospitals
MAXVAR: Strategy of sampling with maximum variation in number of RBCs
RAND: Strategy of random sampling
REGVAR: Strategy of sampling with regional variation
2REG: Strategy of sampling from two regions
